# Resting Energy Expenditure in Anorexia Nervosa: Measured versus Estimated

**DOI:** 10.1155/2012/652932

**Published:** 2011-09-18

**Authors:** Marwan El Ghoch, Marta Alberti, Carlo Capelli, Simona Calugi, Riccardo Dalle Grave

**Affiliations:** ^1^Department of Eating Disorder and Obesity, Villa Garda Hospital, Via Montebaldo, 89, 37016 Garda (VR), Italy; ^2^Department of Neurological, Neuropsychological, Morphological, and Exercise Sciences, University of Verona, 37131 Verona, Italy

## Abstract

*Introduction*. Aim of this study was to compare the resting energy expenditure (REE) measured by the Douglas bag method with the REE estimated with the FitMate method, the Harris-Benedict equation, and the Müller et al. equation for individuals with BMI < 18.5 kg/m^2^ in a severe group of underweight patients with anorexia nervosa (AN). *Methods*. 15 subjects with AN participated in the study. The Douglas bag method and the FitMate method were used to measure REE and the dual energy X-ray absorptiometry to assess body composition after one day of refeeding. *Results*. FitMate method and the Müller et al. equation gave an accurate REE estimation, while the Harris-Benedict equation overestimated the REE when compared with the Douglas bag method. *Conclusion*. The data support the use of the FitMate method and the Müller et al. equation, but not the Harris-Benedict equation, to estimate REE in AN patients after short-term refeeding.

## 1. Introduction

Total energy expenditure (TEE) in humans is determined by resting energy expenditure (REE), dietary-induced thermogenesis, and energy cost of physical activity [1, 2]. REE is the major fraction of TEE in sedentary people, accounting for approximately 70% of TEE [2]. Several studies showed that REE is low in underweight patients with AN compared with normal-weight controls [3–16] and long-term recovered AN patients [17]. The low REE seems mainly due to the loss of lean body mass, a major determinant of REE [18], and to a lesser extent, to the effect of several neuroendocrine changes (e.g., thyroid hormones, adrenal hormones, and leptin) observed in underweight AN patients [19].

The accurate measurement of the REE in underweight AN patients is clinically useful, because it may help us (i) predict the energy level necessary to promote weight restoration and (ii) optimize nutritional rehabilitation preventing severe medical complications such as the refeeding syndrome [20]. Indirect calorimetry, performed with the subject in supine position after an overnight fast, is the most valid method used to measure REE [21]. Unfortunately, this technology is not available in the majority of the hospitals, because it requires skilled technicians and sophisticated methodologies that are costly and difficult to apply in standard clinical settings [16].

Predictive formulas of REE may be used as an alternative to indirect calorimetry that may be utilized by clinicians. The most cited and used predictive formula is the Harris-Benedict equation which includes age, stature, and body weight to estimate REE [22]. Unfortunately, data available on AN patients indicate that Harris-Benedict equation overestimates REE [15, 16, 23, 24]. To overcome this problem, a correction of this equation was derived specifically for individuals with AN and validated in 37 hospitalized female AN patients [24]. However, subsequent studies found that the Schebendach formula is useful for adolescents [25] but seems to underestimate the REE in young adults [16] patients with AN.

A method that might overcome the limits of predictive formulas requires the use of fat-free mass (FFM, kg), and fat mass (FM, kg) to estimate REE. By using this procedure, Müller et al. developed different formulas for different range of body mass index (BMI), including one for a BMI < 18.5 kg/m^2^ [26]. However, this procedure has never been implemented in AN patients by FFM and FM values assessed by means of gold standard body composition methods, such as dual energy X-ray absorptiometry (DXA). 

Recently, advancements in technology have led to the development of relatively inexpensive devices, such as BodyGem [27] and the FitMate [28], designed for estimating REE by measuring only oxygen consumption that might make the use of the indirect calorimetry more popular in clinical settings. Although they have been shown to be accurate when compared with the Douglas bag system [27, 28], the data obtained in severe underweight AN patients are somewhat controversial [29]. For instance, systems for indirect calorimetry that assumed fixed respiratory quotient showed a significant bias with increasing RMR [30]. 

Aim of this study was to compare the results obtained by Douglas bag system in assessing REE with the FitMate method, the Harris-Benedict equation, and the Müller et al. equation for individuals with BMI < 18.5 kg/m^2^ in severe underweight AN patients.

## 2. Methods

### 2.1. Subjects

15 patients (14 females and 1 male) with AN participated in the study. All patients were voluntarily and consecutively admitted to the eating disorder inpatient unit of Villa Garda Hospital during 2010. The patients were referred from all over Italy by general practitioners or by outpatients' eating disorder specialists. Indications for admission were the failure of less intensive treatments (e.g., outpatient treatment) or the presence of an eating disorder of clinical severity not manageable in an outpatient setting. Patients with active substance abuse, schizophrenia, and other psychotic disorders were not included. The indications and contraindications for the inpatient treatment were evaluated during an eligibility interview completed by a senior specialist in the field (RDG). The eating disorder examination interview (EDE) 12.0D [31] was used to generate operational definitions of the DSM-IV diagnoses of AN [32].

Before participation, written informed consent was obtained from all subjects (or by the legal guardian for those less than 18 years old, in accordance with our institution's requirements). The protocol was approved by the Institutional Review Board of Villa Garda Hospital, Verona.

### 2.2. Measurements

Data collection included weight and height measurement, DXA body composition measurement, indirect calorimetry with the Douglas bag and the FitMate methods and Harris-Benedict and the Müller et al. equations.

#### 2.2.1. Body Weight and Height

Body weight was measured on a medical balance and height with a stadiometer by a medical doctor involved in the study. Patients were weighed wearing only underwear and without shoes before breakfast. The BMI was determined according to the usual formula of body weight divided by the squared of height in meters.

#### 2.2.2. Indirect Calorimetry

Indirect calorimetry was performed the second day of admission using the Douglas bag and the FitMate methods in a single session early in the morning before breakfast. The day before the test, all participants consumed with the assistance of a dietitian a diet of 1,750 kcal (protein 21%, carbohydrate 46%, and fat 33%). The order of the two measurements was randomized. Participants were informed to fast overnight, to avoid drinking caffeinated beverages for at least 12 hours, and to abstain from physical activity prior the tests. Upon their arrival in the laboratory, participants rested on a medical bed with the upper part of the body partially raised (+3/4°) and assumed a comfortable position while the instruments were prepared and calibrated and environmental data were recorded. Then, after 10 minutes at rest, the measurements were performed for 11 minutes during which time the participants were instructed to lay quietly, to remain awake and to avoid fidgeting and hyperventilating. 

The Douglas bag method [33] involves collection of the expired air in a large impermeable rubber bag and subsequent volume and analysis of the expired gases [33, 34]. It has been served as the “gold standard” method for many studies in the last decades [28, 35–37]. 

Expired gases were collected by using a mask connected to a two-way, low-resistance respiratory valve whose expiratory outlet was fed to a 100 liter Douglas bag. After gas collection, expired gas composition and volume were assessed using a paramagnetic O_2_ analyser (Oxynos 100, Rosemount Analytical, USA), an infrared CO_2_ meter (Binos 1, Leybold-Heraeus, D), and a dry gas meter (MCS, S.I.M. Brunt, Italy). Gas analysers were calibrated before each experimental trial using gas mixtures of known and certified composition. $\dot{\text{V}}$O_2_ and $\dot{\text{V}}$CO_2_ in STPD conditions were calculated by applying standard equations implying the Haldane correction for inspired ventilatory volume. Respiratory quotient (RQ) was calculated as $\dot{\text{V}}$CO_2_/$\dot{\text{V}}$O_2_ and on the basis of the measured values of RQ, the corresponding value of the energy equivalent of VO_2_ in kJ was calculated. Then, REE was obtained on the basis that 1 kcal equals 4,186 kJ. Finally, the daily REE was calculated by applying a simple proportion. 

The FitMate is a small device designed for measurement of oxygen consumption and energy expenditure during rest and exercise (Cosmed, Rome, Italy). It uses a turbine flow meter for measuring ventilation and a galvanic fuel cell O_2_ sensor for analysing only the fraction of oxygen in expired gases. It is considered to retain the performance of a metabolic cart with a standard mixing chamber or canopy. Sensors measured humidity, temperature, and barometric pressure for use in internal calculations. The FitMate uses standard metabolic formulae to calculate oxygen uptake, and energy expenditure is calculated using a fixed respiratory RQ of 0.85.

#### 2.2.3. Body Composition

 Body composition was assessed by using DXA (iDXA Luner General Electric) in the third day of the admission. No special preparation was required, with the exception that participants wore lightweight clothing for these measures and did not have any metal accessories.

#### 2.2.4. Predictive Formulas of REE

The Harris-Benedict equations [22] for women [655 + (9.6 × weight in kg) + (1.8 × height in cm) − (4.7 × age in years)] and men [(66.47 + (13.75 × weight in kg) + (5.0033 × height in cm) − (6.755 × age in years)], and the Müller et al. equation for individuals with BMI < 18.5 kg/m^2^ [26] [0.08961 × FFM (kg) + 0.05662 × FM (kg) + 0.667] × 238.84 were used to estimate REE.

### 2.3. Statistical Analysis

The Bland-Altman method [38] was used to study the concordance between the Douglas bag method with the FitMate method, and the Harris-Benedict, and Müller et al. equations. The *z*-test was used to evaluate whether the mean of the differences between the values obtained by the three methods, with respect to Douglas bag, was or was not significantly different from zero [39]. The same test was also utilised to evaluate whether the respiratory quotient obtained with Douglas bag was significantly different from the fixed value of 0.85 utilised in the calculations by the FitMate method. Furthermore, the Wilcoxon signed-ranks test was performed to compare the mean REE values of the Douglas bag method with those of the FitMate method, then with those of the Harris-Benedict equation, and finally with those of the Müller et al. equation. Equations and the effect sizes, using the Cohen's d, were calculated [40].

## 3. Results

Table 1 shows the 15 participants' data, with data summarized for age, height, weight, BMI, and REE values. Age ranged from 15 to 45 years, and BMI ranged from 11.96 kg/m^2^ to 16.9 kg/m^2^, with 60% of participants having a BMI < 15 kg/m^2^.

**Figure 1:**
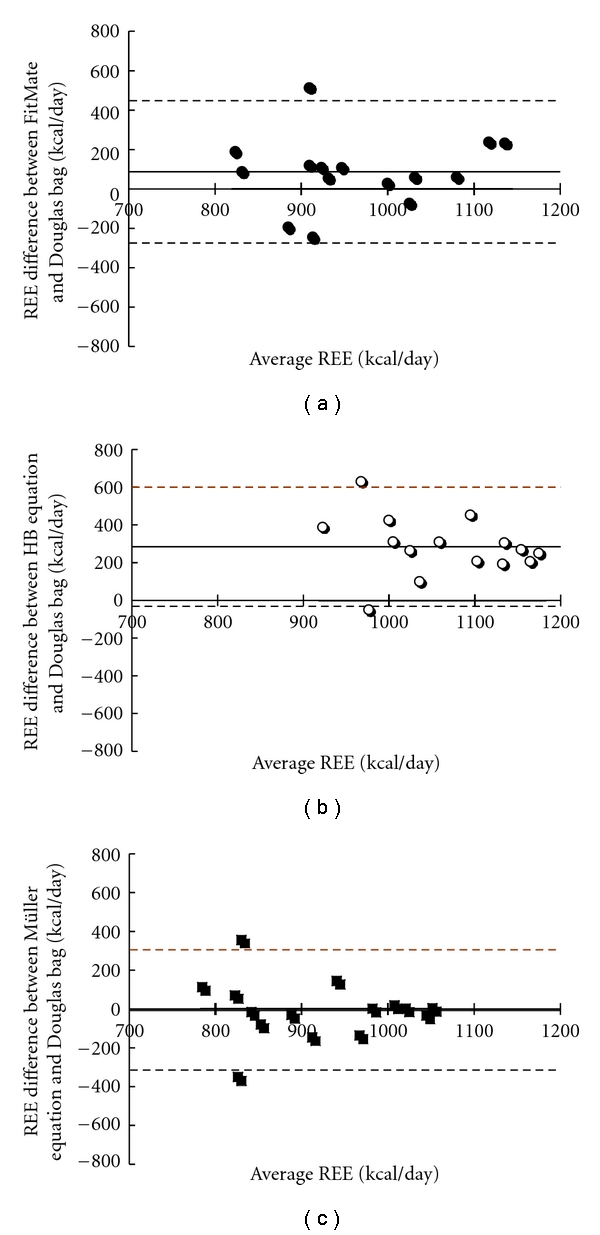
Bland-Altman plots: Difference in the measurements of REE using: (a) FitMate and Douglas bag; (b) Harrison-Benedict equation and Douglas bag; (c) Müller equation and Douglas bag.

Figure 1 shows the Bland-Altman plots reporting the differences between REE values measured with Douglas bag and those obtained by using the other methods (FitMate method and Harris-Benedict and Müller et al. equations from top to bottom). The mean of the differences between the REE values estimated with Harris-Benedict equation and those measured with Douglas bag (bias = 284 kcal/day) was significantly different from zero (6.990; *P* < 0.005). Precision amounted to 158 kcal/day and the 95% limits of agreement ranged from 600 to −30 kcal/day. For the other two comparisons, the means of differences were not significantly different from zero. (FitMate method − Douglas bag method = 1.85; Müller et al. equation − Douglas bag method = −0.125). Bias for the FitMate versus Douglas bags was −87 kcal/day, and precision (SD) turned out to be 181 kcal/day. In turn, the 95% limits of agreement ranged from 448 to −275 kcal/day. As far as the Müller and Douglas Bag comparison is concerned, the values were −5 kcal/day, 155 kcal/day, and 305/−310 kcal/day, for bias, precision, and limits of agreement, respectively.

In Table 2, the data of REE measured with the two systems utilised for IC of RQ obtained with the Douglas bag are reported for each participant in the study. RQ measured with Douglas bag was on the average equal to 0.88 ± 0.07 and turned out to be not significantly different (*P* > 0.3) from 0.85, namely, the fixed value of RQ assumed by FitMate for calculations.

The Wilcoxon signed-ranks test showed no significant differences between the mean REE values estimated with the Douglas bag method and the mean values estimated with the FitMate method (*Z* = −1.70, *P* = 0.088, effect  size = 0.65) and the Müller et al. equation (*Z* = − 0.23, *P* = 0.820, effect  size = 0.04), but significant differences were found comparing mean REE values estimated with the Douglas bag method and with the Harris-Benedict equation (mean REE values: 920.5 ± 124.0 versus 1205.0 ± 99.2, resp., *Z* = − 3.35, *P* = 0.001, effect  size = 2.53).

## 4. Discussion

The principal finding of this study on severe underweight AN patients is that the FitMate method and the Müller et al. equation gave an acceptable REE estimation, while the Harris-Benedict equation overestimated the REE, when compared with the Douglas bag method.

The principal strengths of the study are the use of the Douglas bag method, the DXA, and the EDE interview, three instruments considered the gold standard to assess REE, body composition, and eating disorder diagnosis, respectively. Limitations of the study include the small number of participants, a common problem when studying rare disorder as AN, and the absence of a control group and of longitudinal evaluation. In addition, since patients were tested in a single session, reliability of the single methods for estimating REE was neither evaluated nor quantified.

Participants in the study had severe underweight (mean BMI 14.5 kg/m^2^), marked reduction of FM (mean FM% 9.70), and low REE (mean REE 920.00 kcal/day estimated with Douglas bag method). These data confirm that patients with AN admitted in specialist inpatient units have a condition of severe underweight [41] and, as previously reported [42], a marked loss of FM. They also show that the underweight and the alteration of body composition are associated with an hypometabolic status as consequence of the adaptation to undereating and underweight [19].

Our data confirm that Harris-Benedict equation overestimates the REE in underweight patients with AN [15, 16, 23] and that it should not be used with this population. Using the Harris-Benedict equation to assess the energy need for refeeding underweight AN patients may led to prescribe an excessive energy intake that increase the risk to produce severe negative consequences, such as the refeeding syndrome [20]. This syndrome may include minor complications (e.g., transient pedal edema) or serious complications requiring immediate care (e.g., a prolonged QT interval or hypophosphatemia with associated weakness, confusion, progressive neuromuscular dysfunction, and cardiovascular collapse) [43].

The good news of our data, if confirmed by other studies, is that REE expenditure in underweight AN patients may be estimate with discrete accuracy, after just one day of refeeding, by the FitMate method, and by the Müller et al. equation. The FitMate method, in comparison with the Douglas bag method, is inexpensive, does not requires skilled technicians, and can be used by a wide variety of health professionals to determine the energy need of underweight AN patients. The results reported in the present study confirm and extend those obtained on 60 not underweight healthy adults (*N* = 30 males, *N* = 30 females), where no significant differences between Douglas bag method and FitMate method for oxygen consumption and REE were found [28]. However, the moderate effect size (0.65) of our comparison indicates the need of a bigger sample size to confirm this conclusion.

FitMate utilizes in the calculations a fixed value of RQ set equal to 0.85. Similar devices have shown a poor agreement between REE calculated by assuming a constant and fixed value of RQ (0.85) and that calculated by means of Deltatrac, which was considered wellestablished as a valid and reliable criterion reference system [29]. Specifically, if we do not consider VCO_2_, we are prone to underestimate of REE when RQ is between 0.85 and 1.00 and to overestimate it if RQ is between 0.70 and 0.85 [44]. This might have brought about systematic underestimation of REE in our population, because AN patients seem to be characterized by elevated RQ values larger than 0.85 [29]. However, while previous studies tested participants in severe caloric restriction, our participants have been tested after 24 hours of refeeding. This may explain why the mean RQ of our participants was not significantly larger than 0.85 even though eleven patients out of fifteen had an RQ larger than 0.85. Our data are in agreement with those presented in AN patients after one week of refeeding and characterized by BMI overlapping those of our population [45]. In addition, although FitMate in the present case seemed to overestimate (and not to underestimate) REE, REE values measured with the two approaches were not significantly different. Also, in this case, however, the small sample size, coupled with the inherent inaccuracy of the experimental data, prevents us to draw any clear conclusion and confirm the prediction of the underestimation of REE when assuming a fixed RQ of 0.85 with the FitMate.

The Müller et al. equation is another method that may be used in eating disorder units to estimate REE. This because the DXA, measuring the FM and the FFM required by the Müller et al. equation, is a widely used in eating disorder units to assess the bone mineral density of underweight patients [46].

In conclusion, our data support the use of the FitMate method and the Müller et al. Equation, but not the Harris-Benedict equation, to estimate REE in severe AN patients after short-term refeeding.

## Figures and Tables

**Table 1:** Data of the 15 AN patients (14 females and one male) participating in the study.

	Mean ± SD
*Anthopometrics*	
Height (cm)	162.4 ± 8.74
Weight (kg)	38.4 ± 5.41
BMI (kg/m^2^)	14.5 ± 1.45
Age (yr)	24.8 ± 9.04

*Body composition (DXA)*	
Fat mass (kg)	3.6 ± 2.67
Percent fat mass (%)	9.7 ± 6.27
Fat-free mass (kg)	33.1 ± 5.08
Percent Free fat mass (%)	85.6 ± 5.57

*Resting energy expenditure*	
Douglas bag (kcal/day)	920.5 ± 124.02
FitMate (kcal/day)	1007.1 ± 140.47
Harris-Benedict equation (kcal/day)	1205.0 ± 99.16
Müller et al. equation (kcal/day)	915.1 ± 113.39

Abbreviations: BMI: body mass index; DXA: dual-emission X-ray absorptiometry.

**Table 2:** REE measured in each participant with FitMate and Douglas bag together with individual values of RQ.

Subjects	REE FitMate kcal/day	REE Douglas kcal/day	RQ Douglas
1	1061	1001	0.752
2	788	981	0.869
3	1014	985	0.855
4	1001	892	0.860
5	1252	1019	0.977
6	791	1035	0.873
7	875	787	0.886
8	918	728	0.817
9	1166	652	0.986
10	989	1061	0.823
11	969	849	0.877
12	978	868	0.928
13	959	903	0.843
14	1110	1049	0.932
15	1236	998	1.000
